# Concurrent validity of DorsaVi wireless motion sensor system Version 6 and the Vicon motion analysis system during lifting

**DOI:** 10.1186/s12891-022-05866-w

**Published:** 2022-10-13

**Authors:** Ruth P Chang, Anne Smith, Peter Kent, Nic Saraceni, Mark Hancock, Peter B O’Sullivan, Amity Campbell

**Affiliations:** 1 School of Allied Health, Curtin University, Perth, Australia; 2 Department of Health Professions, Macquarie University, Sydney, Australia; 3 Body Logic Physiotherapy Clinic, Shenton Park, Perth, Australia

**Keywords:** Lumbar spine, Inertial sensor, DorsaVi, Vicon, Concurrent validity

## Abstract

**Background:**

Wearable sensor technology may allow accurate monitoring of spine movement outside a clinical setting. The concurrent validity of wearable sensors during multiplane tasks, such as lifting, is unknown. This study assessed DorsaVi Version 6 sensors for their concurrent validity with the Vicon motion analysis system for measuring lumbar flexion during lifting.

**Methods:**

Twelve participants (nine with, and three without back pain) wore sensors on T12 and S2 spinal levels with Vicon surface markers attached to those sensors. Participants performed 5 symmetrical (lifting from front) and 20 asymmetrical lifts (alternate lifting from left and right). The global-T12-angle, global-S2-angle and the angle between these two sensors (relative-lumbar-angle) were output in the sagittal plane. Agreement between systems was determined through-range and at peak flexion, using multilevel mixed-effects regression models to calculate root mean square errors and standard deviation. Mean differences and limits of agreement for peak flexion were calculated using the Bland Altman method.

**Results:**

For through-range measures of symmetrical lifts, root mean squared errors (standard deviation) were 0.86° (0.78) at global-T12-angle, 0.90° (0.84) at global-S2-angle and 1.34° (1.25) at relative-lumbar-angle. For through-range measures of asymmetrical lifts, root mean squared errors (standard deviation) were 1.84° (1.58) at global-T12-angle, 1.90° (1.65) at global-S2-angle and 1.70° (1.54) at relative-lumbar-angle. The mean difference (95% limit of agreement) for peak flexion of symmetrical lifts, was − 0.90° (-6.80 to 5.00) for global-T12-angle, 0.60° (-2.16 to 3.36) for global-S2-angle and − 1.20° (-8.06 to 5.67) for relative-lumbar-angle. The mean difference (95% limit of agreement) for peak flexion of asymmetrical lifts was − 1.59° (-8.66 to 5.48) for global-T12-angle, -0.60° (-7.00 to 5.79) for global-S2-angle and − 0.84° (-8.55 to 6.88) for relative-lumbar-angle.

**Conclusion:**

The root means squared errors were slightly better for symmetrical lifts than they were for asymmetrical lifts. Mean differences and 95% limits of agreement showed variability across lift types. However, the root mean squared errors for all lifts were better than previous research and below clinically acceptable thresholds. This research supports the use of lumbar flexion measurements from these inertial measurement units in populations with low back pain, where multi-plane lifting movements are assessed.

## Background

Low back pain (LBP) causes more disability than any other health condition and the financial and personal burden attributed to LBP is projected to increase [1, 2]. The role of movement in the development or persistence of LBP remains unclear [3]. There is evidence that people with LBP move differently from people without LBP, with changes such as increased muscle activity (guarding), slower movement and reduced range of movement at the lumbar spine, hip and knee joints previously reported in laboratory settings [4–8]. Reliably and validly measuring lumbar movement in real-life settings has the potential to improve knowledge about the relationship between LBP and movement [9].

One challenge when synthesising the research on lumbar spine movement is the diversity of measurement tools used [10]. In clinical practice, techniques for quantifying lumbar range of movement include: observation, tape measurement, the Schober method, the fingertip-to-floor method and use of tools such as goniometers or inclinometers [11]. Unfortunately, these options are only suitable for assessment of static positions and they lack validity against radiographic ‘gold standard’ measurements [11]. Three-dimensional motion analysis is considered the gold standard in measuring movement, however this equipment is limited to laboratory settings. Because greater movement variation has been shown to occur in daily-living versus laboratory environments, lab-based systems have been criticised for failing to reflect real-life situations [10, 12, 13].

Wearable sensors in the form of inertial measurement units (IMUs) have the potential to accurately measure three-dimensional movement in daily activities outside of the laboratory. These IMUs are small devices comprised of accelerometers, gyroscopes and magnetometers that can be worn for up to 24 h to provide information about movement in real life settings. Some IMUs, such as the DorsaVi system, can also integrate with software to provide real-time biofeedback and capture pain reports (DorsaVi.com, Melbourne, Australia). Their size and portability allow them to be used during a variety of activities and settings, from monitoring activities of daily living [14], to gait analysis [15], and in sporting contexts such as cricket fast-bowling [16]. An earlier version of the DorsaVi sensor showed good concurrent validity with a gold standard 3D system for one dimensional movements of the lumbar spine including flexion, lateral flexion and rotation [17]. Similarly, clinically acceptable agreement has been found between a gold standard 3D system and a Version 5 DorsaVi IMU (DorsaVi.com, Melbourne, Australia) for single plane lumbar movements [9]. In contrast, poorer concurrent validity has been found during complex and faster movements [16, 18]. To date, IMU’s have not been thoroughly tested for their validity during complex movements such as repeated lifting.

Movement sensor technology is evolving rapidly and a completely re-designed DorsaVi IMU, the Version 6, has been available since 2018. This upgraded IMU is 30 × 42 × 8 mm, weighs 12 g and has memory capacity of 256 MB. It uses tri-axial sensors with 100 Hz sampling frequency, ± 2000°/s gyroscope, ± 16G accelerometer, ± 4900µT magnetomter. Concurrent validity between these upgraded DorsaVi Version 6 sensors and reference standard methods, such as Vicon, has not been reported for single plane lumbar movements, nor for any version in multiplane tasks. Therefore, the concurrent validity during lifting tasks from these sensors is unknown. Before these sensors can be implemented in clinical or real-life settings during dynamic tasks, an important first step is understanding their validity relative to a known reference standard. Therefore, the purpose of this study was to determine the concurrent validity of lumbar flexion from DorsaVi Version 6 sensors compared to the Vicon motion analysis system during repeated lifting.

## Methods

### Study design

This study is a concurrent (criterion) validity of DorsaVi Version 6 sensors for measuring lumbar flexion during lifting, using Vicon measurements as the reference standard. Ethical approval was granted from the Human Research Ethics Committee at Curtin University (HRE2018-0197) and written informed consent was obtained.

### Participants

Twelve people from a previous study investigating lumbar and lower limb kinematics and kinetics participated in the current study by wearing DorsaVi Version 6 sensors during a laboratory data collection session [7]. Participants were recruited if they were adults (> 18 years of age) employed in manual handling occupations. Nine participants with, and three without back pain were included. For those with LBP, inclusion criteria were dominant axial LBP (pain between the costal margins and gluteal folds) that was persistent or recurrent for > 3 months duration, with average weekly LBP intensity score of > 3/10, lifting being a primary aggravator of LBP, and an experience of an episode of LBP in the past 12 months that was associated with one of more of the following; work absence, lifting modification, medication use or care-seeking. Exclusion criteria were the presence of acute lumbar radiculopathy, lumbar spine surgery, malignancy or spinal stenosis. For those without LBP, the inclusion criterion was the absence of any episode of disabling LBP in the last 5 years, with an episode defined as having LBP of ≥ 3/10 intensity for more than 24 h which was associated with missed work, activity limitation or care seeking [7].

### Test procedures

**Fig. 1:**
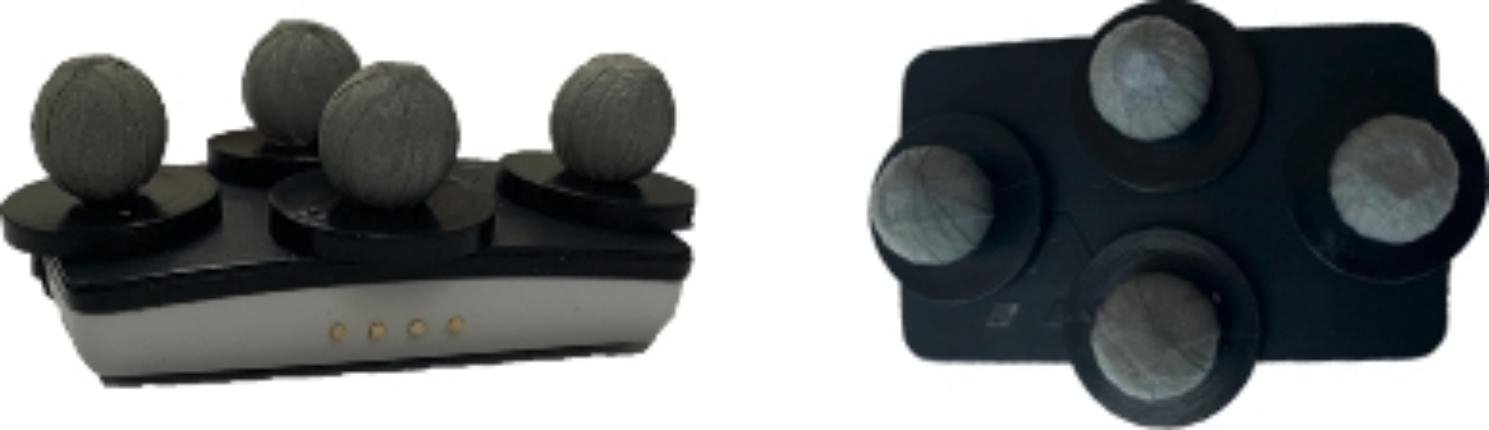
The DorsaVi Version 6 sensor with Vicon markers placed on top (side and top view)

**Fig. 2:**
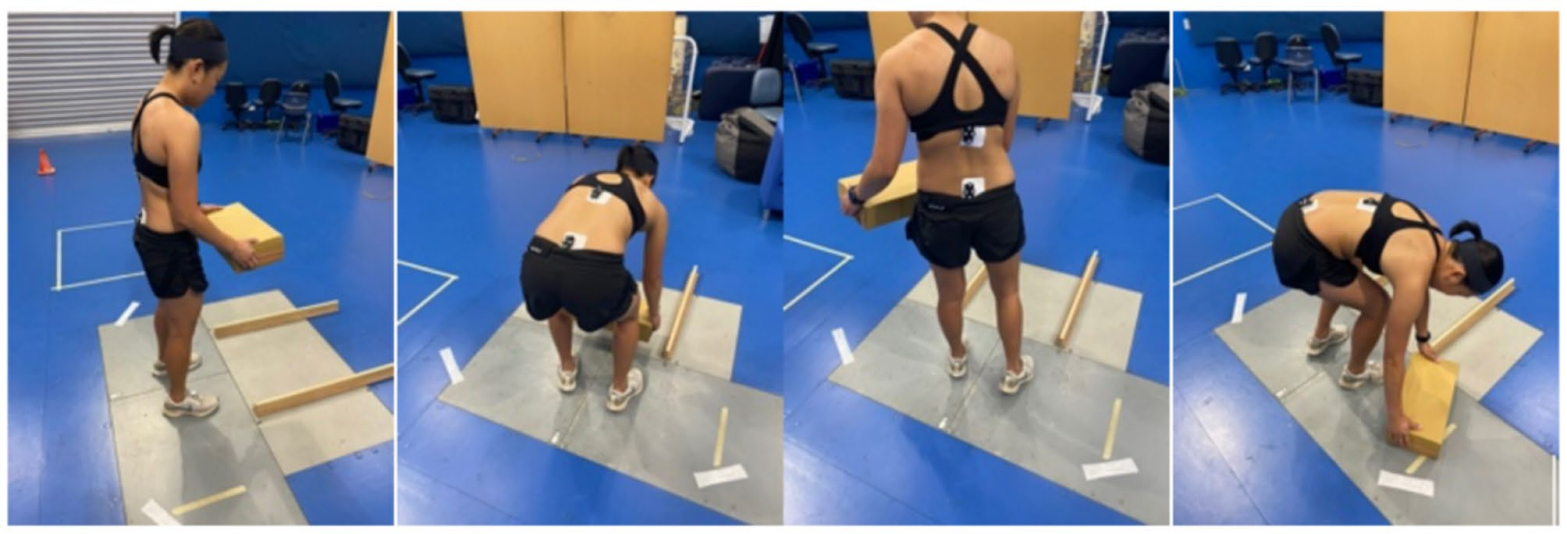
Images of a participant demonstrating the lift positions

Data collection took place at Curtin University motion analysis laboratory. Upon arrival, participants self-completed a questionnaire regarding their demographics. Height and weight were measured by a Physiotherapist using a calibrated stadiometer and scale. With participants standing in a neutral posture, a palpation technique was used by a Physiotherapist with over 10 years’ experience to identify T12 and S2 spinal segments. A systematic review has shown that clinicians can validly and reliably palpate bony landmarks [19]. Patients with back pain were asked to rate their average weekly LBP score using the numerical pain rating scale (NPRS) which ranges from 0 (no pain) to 10 (worst imaginable). The NPRS is suitable for representing clinically meaningful change in LBP populations [20]. Data for this study was collected using the 19-camera Vicon MX motion analysis system (Oxford metrics Inc., Oxford, UK) (250 Hz) and two Version 6 IMU sensors (DorsaVI.com, Melbourne, Australia). The IMUs were placed on T12 and S2 spinal segments using double-sided tape (3 M foam mounting tape, 3 M Minnesota, USA). The Vicon markers fixed to the outer surface of the sensors to minimise soft tissue artefact (Fig. 1) [21, 22]. This procedure was similar to that used in previous studies [9, 17]. The lifting task comprised of a series of 25 lifts (5 symmetrical and 20 asymmetrical) with an empty box (200 g). The first five lifts were symmetrical lifts where the participant lifted a box placed directly in front of them, while the 20 asymmetrical lifts involved the participant alternating lifting a box from their left or right (Fig. 2). All lifts were from the floor and participants were encouraged to lift in whatever way they normally would [7].

### Definition of measurement angles

The DorsaVi Version 6 system calculated the angle of the upper sensor (global-T12-angle) and lower sensor (global-S2-angle) separately, relative to the line of gravity. The angle between these two global sensors was calculated as the ‘relative-lumbar-angle’. All three measures were reported for lumbar flexion in the sagittal plane (flexion, extension) and are reported in the results.

### Data Processing

The Vicon data were processed using Vicon Nexus motion analysis software (Vicon, Oxford Metrics, Oxford, UK) and filtered using a fourth-order low-pass Butterworth filter operating at a cut-off frequency of 2 Hz, a threshold determined using a residual analysis [7]. The Vicon data were output and available in a Microsoft Excel format. DorsaVi software (MDMv6 Manager v6.883, DorsaVi.com) was used to import the raw data from the sensors onto a computer, and inbuilt Kalman filters were used to estimate the orientation of the sensors relative to a global fixed coordinate system. These data were processed using an online file reader (DorsaVi Data Processor v1.0.0) and output in Microsoft Excel format. A custom Lab-VIEW program (National Instruments, Texas, USA) down-sampled the Vicon data to 100 Hz to allow direct comparison with DorsaVi data, and both datasets were time-synchronised to generate 101 data points per lift. The interquartile range (IQR) method of outlier detection was used, whereby outliers were identified as being those more than 1.5 IQR below Q1 or more than 1.5 IQR above Q3 [23]. When present, the outlier data were removed from analysis. These data were assembled for input into statistical software (Stata/BE 17.0 for Mac, Statacorp, College Station, Texas, USA) for further analysis.

### Statistical analysis

Descriptive data about the study sample were presented as means (95% CI). To assess concurrent validity of the DorsaVi Version 6 sensors, the differences between DorsaVi Version 6 and Vicon sensors peak flexion and through-range measures were calculated. Differences between DorsaVi Version 6 and Vicon were estimated by calculating the root mean square error (RMSE) with accompanying 95% confidence interval between measurement methods by use of a multilevel mixed-effects linear regression model, where the DorsaVi Version 6 data were the independent variable and the Vicon data the dependent variable [9]. Agreement between DorsaVi Version 6 and Vicon for peak flexion measures was also quantified using Bland Altman plots of the mean difference and 95% limits of agreement (LOA) to represent the random error between methods [24].

## Results

Nine people with LBP and three people without LBP participated in this study. Their demographic data are presented in Table 1. The LBP participants’ average pain score was 4 out of 10.

**Table 1:** Description of participants/study sample

Characteristics	All participants (n = 12)		
	Mean (SD)	95% CI	Min to max range
Age (yr)	35.3 (15.6)	25.4 to 45.2	19 to 66
Female Back pain Weight (kg)	3 (25%) 9 (75%) 75.0 (14.2)	66.0 to 84.0	55 to 100
Height (cm)	177.1 (12.0)	169.6 to 184.7	157 to 193
BMI (kg/m^2^)	23.7 (2.5)	22.2 to 25.3	18.7 to 26.9

*BMI =* body mass index

After outliers were excluded, 96–100% of data remained. The number of participants, observations, lifts and the number of observations per participant analysed are detailed in Table 2. The RMSEs by lifting type and angle for peak flexion and through-range measures are presented in Table 3. For through-range measures during symmetrical lifts, RMSEs varied between 0.86° and 1.34°, and were highest for relative-lumbar-angle. In contrast, RMSEs were similar for through-range measures during asymmetrical lifts, ranging from 1.7° and 1.9°. For symmetrical lifts, peak flexion RMSEs were between 0.44° and 0.68°, and for asymmetrical lifts peak flexion RMSEs were between 1.24° and 2.06°

**Fig. 3:**
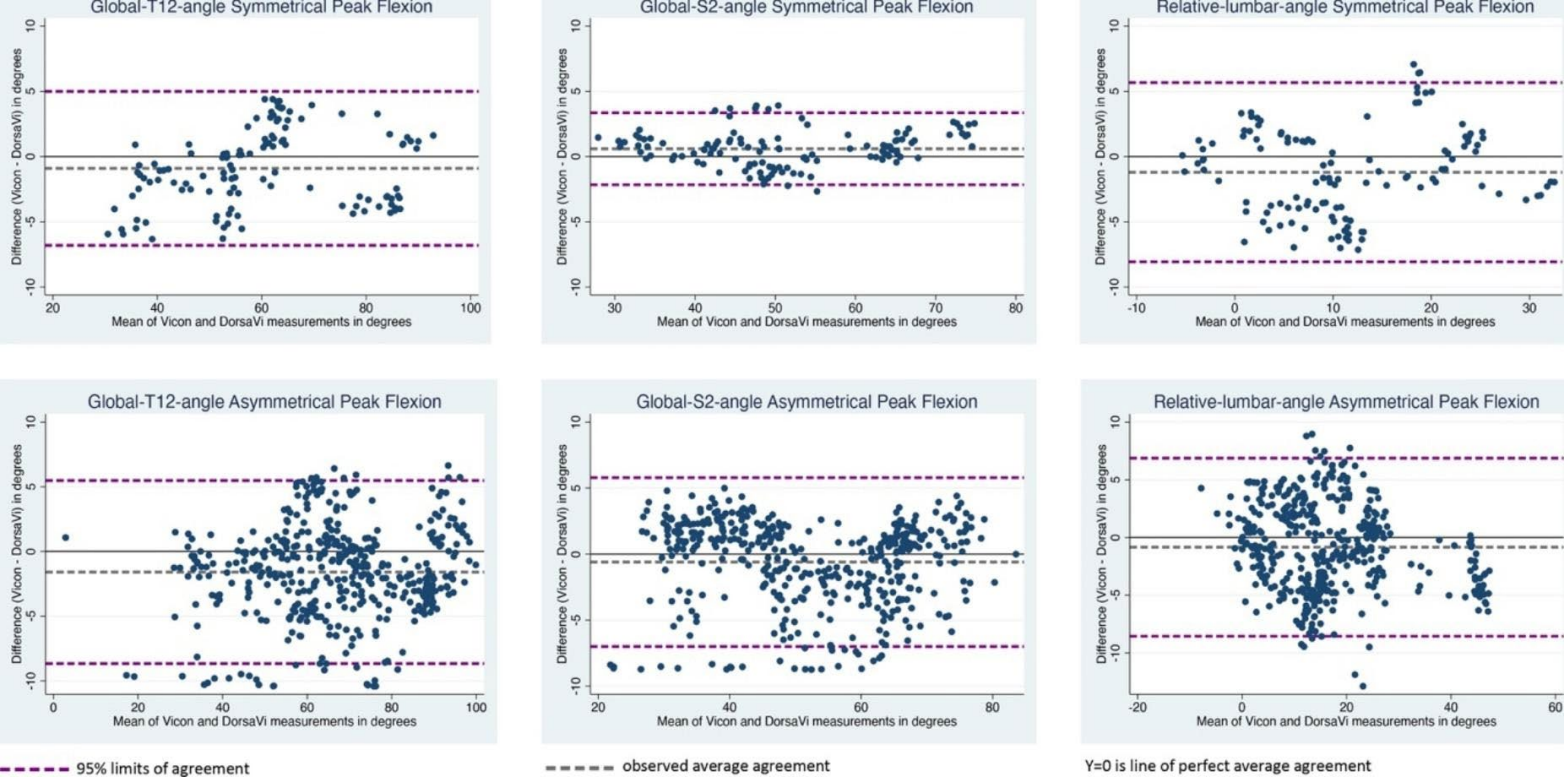
**Bland Altman Peak Flexion Plots** showing the range within which 95% of the differences in DorsaVi Version 6 and Vicon system measurements occurred

The mean differences and LOA for peak flexion measures are presented in Table 3. Mean difference was between − 1.20° and 0.60° for symmetrical lifts, and between − 1.59° and − 0.60° for asymmetrical lifts. The 95% LOA for the peak flexion measures showed variation across lift type and measures (Fig. 3; Table 3) with the narrowest being − 2.16° to 3.36° for the global-S2-angle sensor during symmetrical lifts, and the widest − 8.55° to 6.88° for the relative-lumbar-angle angle for asymmetrical lifts

**Table 2:** The number of participants, observations, lifts and observations per participant analysed

Flexion	Number of participants	Number of observations	Number of lifts (Min to max range)	Number of observations/participant (Min to max range)
**Symmetrical Peak Flexion**				
Global-T12-angle	12	120	5 (5 to 5)	10 (10 to 10)
Global-S2-angle	12	120	5 (5 to 5)	10 (10 to 10)
Relative-lumbar-angle	12	120	5 (5 to 5)	10 (10 to 10)
**Asymmetrical Peak Flexion**				
Global-T12-angle	12	440	14.8 (6 to 25)	36.7 (32 to 40)
Global-S2-angle	12	439	14.8 (6 to 25)	36.6 (32 to 41)
Relative-lumbar-angle	12	440	14.8 (6 to 25)	36.7 (32 to 40)
**Symmetrical Through-Range**				
Global-T12-angle	12	6,047	3 (1 to 5)	504 (492 to 505)
Global-S2-angle	12	5,921	3 (1 to 5)	493 (391 to 505)
Relative-lumbar-angle	12	6,060	3 (1 to 5)	505 (505 to 505)
**Asymmetrical Through-Range**				
Global-T12-angle	12	21,300	14.8 (6 to 25)	1,775 (1,338 to 2,018)
Global-S2-angle	12	21,569	14.8 (6 to 25)	1,797 (1,562 to 2,020)
Relative-lumbar-angle	12	22,204	14.8 (6 to 25)	1,850 (1,616 to 2,020)

**Table 3:** Peak and through-range measurement differences between DorsaVi Version 6 and Vicon systems

Flexion	Root mean square error (SD)	95% CI	Mean difference	Lower limit of agreement	Upper limit of agreement
**Symmetrical Peak Flexion**					
Global-T12-angle	0.55 (± 0.49)	0.46–0.64	-0.90	-6.80	5.00
Global-S2-angle	0.44 (± 0.34)	0.38–0.50	0.60	-2.16	3.36
Relative-lumbar-angle	0.68 (± 0.55)	0.58–0.78	-1.20	-8.06	5.67
**Asymmetrical Peak Flexion**					
Global-T12-angle	2.06 (± 1.59)	1.91–2.21	-1.59	-8.66	5.48
Global-S2-angle	2.00 (± 1.63)	1.85–2.15	-0.60	-7.00	5.79
Relative-lumbar-angle	1.24 (± 1.08)	1.14–1.34	-0.84	-8.55	6.88
**Symmetrical Through-Range**					
Global-T12-angle	0.86 (± 0.78)	0.84–0.88			
Global-S2-angle	0.90 (± 0.84)	0.88–0.92			
Relative-lumbar-angle	1.34 (± 1.25)	1.31–1.37			
**Asymmetrical Through-Range**					
Global-T12-angle	1.84 (± 1.58)	1.82–1.86			
Global-S2-angle	1.90 (± 1.65)	1.88–1.92			
Relative-lumbar-angle	1.70 (± 1.54)	1.68–1.72			

## Discussion

This is the first study to assess concurrent validity between the DorsaVi Version 6 system and the Vicon motion analysis system for lumbar spine angles, and the first to assess this during both symmetrical and asymmetrical lifting. The results showed RMSE of 0.86° to 1.90° for through-range measures and 0.55° to 2.06° for peak flexion. Values for mean differences between peak flexion from the two systems were ≤ 1.59°. For peak flexion, the lowest RMSE was at the global-S2-angle during symmetrical lifting (0.44° ± 0.34), and the highest was at global-T12-angle in asymmetrical lifting (2.06° ± 1.59). For through-range measures, the lowest RMSE was at global-T12-angle during symmetrical lifting (0.86° ± 0.78), and highest was at global-S2-angle during asymmetrical lifting (1.90° ± 1.65). Only global-S2-angle during symmetrical lifting had 95% LOAs less than 5° (-2.16° to 3.36°), with all other limits greater than 5°, with the highest being for relative-lumbar-angle during asymmetrical lifting (-8.55° to 6.88°).

Unsurprisingly, symmetrical lifting demonstrated smaller RMSEs for through-range (0.86° to 1.34°) and peak flexion measures (0.44° to 0.68°) when compared to asymmetrical lifting through-range (1.7°-1.9°) and peak flexion measures (1.2° to 2.1°). These values are smaller compared to RMSE values reported for the previous DorsaVi Version 5 sensor for single plane lumbar flexion through-range (1.82°) [9]. The results of our study support previous research that has shown errors are generally task dependent, occurring when movements are complex, multi-plane and at extremes of end range [16, 17, 25, 26]. However, differences in RMSEs between symmetrical and asymmetrical lifting were small, with the maximum difference being for peak flexion at the S2 sensor (1.56°).

Determining concurrent validity by estimating LOA relative to a reference system is uncommon in concurrent validity studies using sensors, but provides useful information as it contains the difference between measurements by both systems for 95% of future measurement pairs. Our 95% LOA varied considerably across all measures and all ranges crossed the zero point for each measure indicating an absence of systematic bias. The widest LOA was seen in the relative-lumbar-angle for both symmetrical (-8.1° to 5.7°) and asymmetrical (-8.6° to 6.9°) peak flexion. Only one study has previously reported LOA for lumbar flexion (-3.86° to 4.69°) [9]. The wider LOA demonstrated in our study may be attributed to differences in software or hardware between DorsaVi Version 5 and 6 sensors or the multiplane lifting task examined.

Despite some previous research suggesting an acceptable LOA of < 5° for concurrent validity [9, 25, 27], these suggested values are only based on systematic reviews that have found the majority of studies report RMSE or SD between 2° and 5° [28–30]. However, there is little consistency in reporting of errors in the existing literature with some studies reporting absolute error, mean difference, RMSE, correlation coefficients and coefficients of determination. Because comparability is difficult between such a variety of measurement approaches, rather than a statistical decision, identifying appropriate LOA remains a clinical decision about specific applications [31]. It is useful to compare other methods of clinical assessment of movement to determine whether our LOA variation as measured by an IMU seems reasonable for clinical practice. Clinically, one method of measuring lumbar movement is to use an inclinometer. Differences in measures of lumbar spine ROM made by an inclinometer with and without fluoroscopy assistance have standard deviations < 15.6° [32]. Lumbar spine mobility measured with a single and double inclinometer, reported median measurement error to be 8.5° and 10.5° respectively [33]. Considering these large measurement error variations, and the practical difficulties in using an inclinometer to measure dynamic activities such as lifting, the LOA reported in our study suggest that IMUs have acceptably small errors compared to the gold standard of laboratory-based motion capture and the DorsaVi Version 6 sensors are therefore appropriate for measuring lumbar flexion during lifting in clinical practice.

Although many studies have looked at the performance of commercial IMU systems compared to gold-standard 3D methods in other body regions, few concurrent validity studies have measured the lumbar spine and none have measured both through-range and peak flexion measures. Having both through-range and peak flexion measures has the potential to provide additional information about movement, such as coordination, speed and timing of movement [9]. Knowing the level of error in both measures may assist clinicians in appropriately interpreting these subtle movement characteristics.

Our through-range RMSE values of < 1.7° to 1.9° were better than those reported in previous studies of lumbar flexion during forward bending (1.8° to 4.1°), the single plane movement most comparable to lifting [9, 34]. Two concurrent validity studies have reported RMSEs for end range or peak values. A study by Charry and colleagues [17] compared an early version IMU with a 3D lab-based system in single and multi-plane lumbar spine movements and found end range errors increased by < 2.3° for flexion in multiplane movements. A study that compared trunk movements in everyday activities between three inertial sensor modules and the 3D Vicon system found peak value RMSE (∼2.5 deg) occurred in flexion [35]. In contrast, our study is based on a comparatively large sample and larger number of data points, and showed asymmetrical peak flexion RMSE being < 1.62° larger when compared to symmetrical lift RMSE. This demonstrates a small increase in RMSE of flexion between asymmetrical and symmetrical lifting. Our RMSE results for through-range and peak flexion measures in DorsaVi Version 6 sensors perform comparatively well or better than existing results.

Several systematic reviews have investigated IMU accuracy for upper and lower limb joints [28, 29, 36]. A recent review of criterion validity of IMUs by Poitras and colleagues [29], found trunk movement studies have been mostly performed using closed-chain single-plane movement, and have provided better RMSE results than those of the shoulder, hip, knee or ankle. Poitras and colleagues [29] reported RMSE for IMU validity when compared to a gold standard between 1.8° and 5.9° from nine trunk studies, 0.2° and 11.8° from 13 hip studies, 1° to 11.5° from 15 knee studies, 0.4° to 18.8° from 11 ankle studies. These RMSE results reinforce that DorsaVi Version 6 sensors performed similarly well during lifting. It is beyond the scope of this paper to hypothesise if differences in measurement error across studies arose from differences between custom and commercial sensor systems, the sensor software/hardware or study design differences.

We reported two global angles, (global-T12-angle and global-S2-angle) along with a relative measure, (relative-lumbar-angle) in order to investigate whether the three different measures provided differences in measurement error. There were no consistent differences between these global and relative measures and differences in RMSE across all measures varied between 0.20° and 0.82°. Because these differences in RMSE are so similar, we expect clinicians would preferentially use relative-lumbar-angle as it seems to more closely represent lumbar spine movement.

Clinicians commonly assess movement with visual analysis to identify patterns of dysfunction and to monitor change [5, 37]. However, visual assessment of movement quality has been shown to vary amongst physiotherapists with differing clinical experience [38], and may only be accurate for changes of 12 degrees or more of movement range in single-please, low-speed movements [39]. Our results demonstrate that the DorsaVi Version 6 system provides an alternative tool to visual assessment or the use of inclinometers, and can provide clinically acceptable lumbar flexion measurements during symmetrical and asymmetrical lifting tasks. These wearable sensors have the ability to quantify general activity levels and may also provide insight into how people may move when away from the clinic, capturing movement for continuous periods of time in real-life settings such as workplaces or home. Collectively, this information may potentially assist in capturing information regarding movement before pain develops, or as symptoms improve.

## Strengths and limitations

Strengths of this study included a clinically relevant sample that included participants with and without back pain, as that reflects a clinical population at variable stages of recovery. In the systematic review by Papi and colleagues [10], only five out of 22 papers included a clinical population. We also included a clinically relevant task (lifting) that is often reviewed by clinicians, when many previous studies have only investigated single plane ROM tasks. Previous literature also shows diverse measurement tools and outcomes. We opted to provide both peak flexion and through-range measures at two global and a relative angle to provide a thorough comparison to previous literature, but also to increase the likelihood that we captured any potential measurement differences. Our chosen statistical approach is suitable for measuring agreement with consideration for repeated observations and variability within different levels of data, minimising the risk of inappropriately narrow LOA [24]. Finally, errors that result from sensor malpositioning, poor fixation and incorrect identification of landmarks were minimised, but would apply to both the measurement systems equally, so are therefore unlikely to have changed the results of this study. Only three participants without low back pain were included in this study, and further studies with larger sample sizes could provide greater insight into the concurrent validity between DorsaVi Version 6 sensors and the Vicon motion analysis system in this population. A limitation of this study was that we only analysed measurement error in the sagittal plane. Analysis of lateral flexion or rotation during multiplane tasks may provide further insight into measurement error of IMUs during complex movements.

## Clinical implications

The results of this study provide preliminary evidence that the DorsaVi Version 6 system provides acceptable concurrent validity with the Vicon measurement system in symmetrical and asymmetrical lifting for people with and without LBP. The size, relatively low cost and portability of IMU sensors make them an attractive option for clinicians interested in assessing or providing biofeedback during functional activities such as lifting. A major benefit is that IMUs can potentially provide lumbar flexion measurements of lifting in a range of contexts, from clinical environments to real-world settings. The ability to provide feedback across contexts may potentially have implications for work-place assessment and management of lifting-associated conditions. However, before it is possible to confidently embrace this technology it is important to validate these IMU systems across many commonly assessed tasks.

## Conclusion

This study found clinically acceptable concurrent validity between the DorsaVi Version 6 system and the Vicon measurement system in symmetrical and asymmetrical lifting for people with and without LBP. This research supports the use of lumbar flexion measurements from IMUs in situations where multi-plane lifting movements are assessed. Further research should investigate other complex multiplane movements in all movement planes to allow clinicians to better understand concurrent validity and to better understand the use of IMUs in broader contexts and across different activities.

## Data Availability

Raw data are available in Excel spreadsheet format upon request. Requests can be made to Ruth Chang (ruth.chang@postgrad.curtin.edu.au).

## List of abbreviations

LBP: Low back pain
IMUs: Inertial measurement units
Hz: Hertz
95% CI: 95% confidence interval
LOA: Limits of agreement
RMSE: Root mean square error

## References

[1] Wu A, March L, Zheng X, Huang J, Wang X, Zhao J (2020). Global low back pain prevalence and years lived with disability from 1990 to 2017: estimates from the Global Burden of Disease Study 2017. Ann Transl Med.

[2] Hartvigsen J, Hancock MJ, Kongsted A, Louw Q, Ferreira ML, Genevay S (2018). What low back pain is and why we need to pay attention. Lancet.

[3] Christe G, Aussems C, Jolles BM, Favre J (2021). Patients with chronic low back pain have an individual movement signature: A comparison of angular amplitude, angular velocity and muscle activity across multiple functional tasks. Front Bioeng Biotechnol.

[4] Laird RA, Keating JL, Kent P (2018). Subgroups of lumbo-pelvic flexion kinematics are present in people with and without persistent low back pain. BMC Musculoskelet Disord.

[5] Laird R, Gilbert J, Kent P, Keating J (2014). Comparing lumbo-pelvic kinematics in people with and without back pain: A systematic review and meta-analysis. BMC Musculoskelet Disord.

[6] Tsang SMH, Szeto GPY, Li LMK, Wong DCM, Yip MMP, Lee RYW (2017). The effects of bending speed on the lumbo-pelvic kinematics and movement pattern during forward bending in people with and without low back pain. BMC Musculoskelet Disord.

[7] Saraceni N, Campbell A, Kent P, Ng L, Straker L, O’Sullivan P (2021). Exploring lumbar and lower limb kinematics and kinetics for evidence that lifting technique is associated with LBP. PLoS ONE.

[8] Hernandez A, Gross K, Gombatto S (2017). Differences in lumbar spine and lower extremity kinematics during a step down functional task in people with and people without low back pain. Clin Biomech Elsevier Ltd.

[9] Mjøsund HL, Boyle E, Kjaer P, Mieritz RM, Skallgård T, Kent P (2017). Clinically acceptable agreement between the ViMove wireless motion sensor system and the Vicon motion capture system when measuring lumbar region inclination motion in the sagittal and coronal planes. BMC Musculoskelet Disord.

[10] Papi E, Koh WS, McGregor AH (2017). Wearable technology for spine movement assessment: A systematic review. J Biomech.

[11] Littlewood C, May S (2007). Measurement of range of movement in the lumbar spine—what methods are valid? A systematic review. Physiotherapy.

[12] Nishi Y, Shigetoh H, Fujii R, Osumi M, Morioka S (2021). Changes in trunk variability and stability of gait in patients with chronic low back pain: Impact of laboratory versus daily-living environments. J Pain Res.

[13] Dreischarf M, Pries E, Bashkuev M, Putzier M, Schmidt H (2016). Differences between clinical “snap-shot” and “real-life” assessments of lumbar spine alignment and motion – What is the “real” lumbar lordosis of a human being?. J Biomech.

[14] Nguyen H, Lebel K, Bogard S, Goubault E, Boissy P, Duval C (2018). Using inertial sensors to automatically detect and segment activities of daily living in people with Parkinson’s Disease. IEEE Trans Neural Syst Rehabil Eng.

[15] Seel T, Raisch J, Schauer T (2014). IMU-based joint angle measurement for gait analysis. Sensors.

[16] Cottam DS, Campbell AC, Davey MPC, Kent P, Elliott BC, Alderson JA (2022). Measurement of uni-planar and sport specific trunk motion using magneto-inertial measurement units: The concurrent validity of Noraxon and Xsens systems relative to a retro-reflective system. Gait Posture.

[17] Charry E, Umer M, Taylor S, editors. Design and validation of an ambulatory inertial system for 3-D measurements of low back movements. 2011 Seventh International Conference on Intelligent Sensors, Sensor Networks and Information Processing; 2011 on: 6–9 Dec. 2011.

[18] Lebel K, Boissy P, Hamel M, Duval C (2013). Inertial measures of motion for clinical biomechanics: comparative assessment of accuracy under controlled conditions - effect of velocity. PLoS ONE.

[19] Adhia DB, Bussey MD, Ribeiro DC, Tumilty S, Milosavljevic S (2013). Validity and reliability of palpation-digitization for non-invasive kinematic measurement - a systematic review. Man Ther.

[20] Childs JD, Piva SR, Fritz JM (2005). Responsiveness of the numeric pain rating scale in patients with low back pain. Spine (Phila Pa 1976).

[21] Zhang J-T, Novak AC, Brouwer B, Li Q (2013). Concurrent validation of Xsens MVN measurement of lower limb joint angular kinematics. Physiol Meas.

[22] Ha T-H, Saber-Sheikh K, Moore AP, Jones MP (2012). Measurement of lumbar spine range of movement and coupled motion using inertial sensors – A protocol validity study. Man Ther.

[23] Tukey J. Exploratory data analysis. Reading, Mass.: Reading, Mass. Addison-Wesley Pub. Co.; 1977.

[24] Bland JM, Altman DG (2007). Agreement between methods of measurement with multiple observations per individual. J Biopharm Stat.

[25] O’Sullivan K, O’Sullivan L, Campbell A, O’Sullivan P, Dankaerts W (2012). Towards monitoring lumbo-pelvic posture in real-life situations: Concurrent validity of a novel posture monitor and a traditional laboratory-based motion analysis system. Man Ther.

[26] Robert-Lachaine X, Mecheri H, Larue C, Plamondon A (2016). Validation of inertial measurement units with an optoelectronic system for whole-body motion analysis. Med Biol Eng Comput.

[27] Intolo P, Carman AB, Milosavljevic S, Abbott JH, Baxter GD (2009). The Spineangel®: Examining the validity and reliability of a novel clinical device for monitoring trunk motion. Man Ther.

[28] Cuesta-Vargas AI, Galán-Mercant A, Williams JM (2010). The use of inertial sensors system for human motion analysis. Phys Therapy Reviews.

[29] Poitras I, Dupuis F, Bielmann M, Campeau-Lecours A, Mercier C, Bouyer LJ (2019). Validity and reliability of wearable sensors for joint angle estimation: A systematic review. Sensors.

[30] McGinley JL, Baker R, Wolfe R, Morris ME (2009). The reliability of three-dimensional kinematic gait measurements: A systematic review. Gait Posture.

[31] Bland JM, Altman DG (1986). Statistical methods for assessing agreement between two methods of clinical measurement. Lancet.

[32] Saur PMM, Ensink F-BM, Frese K, Seeger D, Hildebrandt J (1996). Lumbar range of motion: Reliability and validity of the inclinometer technique in the clinical measurement of trunk flexibility. Spine (Phila Pa 1976).

[33] Rondinelli R, Murphy J, Esler A, Marciano T, Cholmakjian C (1992). Estimation of normal lumbar flexion with surface inclinometry: A comparison of three methods. Am J Phys Med Rehabil.

[34] Bauer CM, Rast FM, Ernst MJ, Kool J, Oetiker S, Rissanen SM (2015). Concurrent validity and reliability of a novel wireless inertial measurement system to assess trunk movement. J Electromyogr Kinesiol.

[35] Wong WY, Wong MS (2008). Trunk posture monitoring with inertial sensors. Eur Spine J.

[36] Walmsley CP, Williams SA, Grisbrook T, Elliott C, Imms C, Campbell A (2018). Measurement of Upper Limb Range of Motion Using Wearable Sensors: A Systematic Review. Sports Med - Open.

[37] Kent PM, Keating JL, Taylor NF (2008). Primary care clinicians use variable methods to assess acute nonspecific low back pain and usually focus on impairments. Man Ther.

[38] Whatman C, Hing W, Hume P (2012). Physiotherapist agreement when visually rating movement quality during lower extremity functional screening tests. Phys Ther Sport.

[39] Abbott E, Campbell A, Wise E, Tidman SJ, Lay BS, Kent P (2022). Physiotherapists could detect changes of 12 degrees or more in single-plane movement when observing forward bending, squat or hand-over-head: A cross-sectional experiment. Musculoskelet Sci Pract.

